# The effect of conventional and ohmic heating as pasteurization methods on the mechanical and rheological properties of edible whey‐based films

**DOI:** 10.1111/1750-3841.70180

**Published:** 2025-04-04

**Authors:** Elvidas Aleksandrovas, Agnė Vasiliauskaitė, Jorge M. Vieira, Joana T. Martins, Ricardo N. Pereira, Antonio A. Vicente, Vitalijs Radenkovs, Ida Rud, Mindaugas Malakauskas, Loreta Šernienė

**Affiliations:** ^1^ Department of Food Safety and Quality, Veterinary Academy Lithuanian University of Health Sciences Kaunas Lithuania; ^2^ Centre of Biological Engineering (CEB) University of Minho, Campus de Gualtar Braga Portugal; ^3^ LABBELS—Associate Laboratory Braga/Guimarães Portugal; ^4^ Research Laboratory of Biotechnology, Division of Smart Technologies Latvia University of Life Sciences and Technologies Jelgava Latvia; ^5^ Institute of Horticulture (LatHort) Dobele Latvia; ^6^ Fisheries and Aquaculture Research Norwegian Institute of Food, Nofima Ås Norway

**Keywords:** acid whey permeate, conventional heating, edible films, liquid acid whey protein concentrate, ohmic heating

## Abstract

Edible film‐forming solutions typically undergo thermal treatment to ensure microbial safety before being applied to food products. The aim of this study was to assess the effects of two different heating methods—conventional heating (CH) and ohmic heating (OH)—on the physical, chemical, and microbiological properties of liquid acid whey permeate (AWP) and liquid acid whey protein concentrate (AWPC) edible films. Composition of edible film‐forming solutions consisted of AWPC, sunflower oil, sugar beet pectin, and glycerol, whereas AWP‐based films were produced with sugar beet pectin and glycerol. The following parameters were tested to assess the effect of heating treatments on the film‐forming solutions: rheology, contact angle [CA] and microbial counts and mechanical properties (tensile strength [TS] and elongation at break [EB]), water vapor permeability [WVP], moisture content [MC], solubility (Sol), and thickness with optical properties of produced edible films. In addition, film surface was investigated by scanning electron microscopy [SEM]. Microbiological analysis of the untreated film‐forming solutions revealed that the AWPC‐based solution had a higher initial load of lactic acid bacteria (3.96 log_10_ CFU/mL) (*p* < 0.05). Both heating treatments successfully reduced microbial counts to below detection limits in both film‐forming solutions. Additionally, OH treatment resulted in lower CA values in both solutions (*p* < 0.05). OH also led to an increase in TS for AWP‐based edible films (*p* < 0.05) and significantly reduced the thickness of both AWP and AWPC films, while reducing the Sol of AWP‐based films and increasing the Sol of AWPC‐based films (*p* < 0.05). The study highlights the effectiveness of the two pasteurization methods and offers insights into improving whey‐based edible films.

## 1 INTRODUCTION

Biodegradable materials, specially made from second‐hand substances, have drawn much attention in recent years due to increased awareness of consumers with environmental aspects (Enujiugha & Oyinloye, 2019). One type of packaging material that is used in the food industry is biodegradable edible films, which are described as thin sheets that can be placed on food that ensure barrier properties, nutritional quality preservation, and safety and bring functionality to the product and present intrinsic benefits compared to synthetic films (Bizymis & Tzia, 2022). To determine whether a film is edible or non‐edible, several factors must be considered. Edible films must be composed of food‐grade ingredients that meet regulatory standards and are made from substances recognized as generally recognized as safe. In contrast, non‐edible films are primarily used for packaging and external protection, often made from synthetic, non‐edible materials such as plastic, and are not intended for consumption (Marsh & Bugusu, 2007; Suput et al., 2015). The most common benefit of biodegradable edible films is that once they are applied to a food product, they become part/components of it and can be consumed together with food without requiring to be removed or discarded (Shanbhag et al., 2023).

Edible films are made from various substances derived from a variety of agricultural commodities. In recent years, food industry‐derived waste has become of interest for edible film development. The most common materials used in the production of edible films are lipids, polysaccharides, and proteins. Specifically, whey (a dairy industry waste) could become a good material for edible film production (Jiang et al., 2020). There are two types of whey: (i) acid whey, a byproduct of cottage cheese and Greek yogurt production (pH < 5) and (ii) sweet whey, a byproduct of cheese and yogurt production (pH 6–7) based on the method used to precipitate the casein. The whey ultrafiltration results in a concentrated liquid of proteins that could further be dried and produced as whey protein concentrate powder. In small dairy factories, acid whey is usually disposed of and results in environmental damage. Thus, bringing whey back into the production process could be beneficial (Menchik et al., 2019; Wherry et al., 2019).

The process to produce an edible film requires a few steps based on the materials used and involves (i) a structured biopolymer that forms a matrix; (ii) enhancers of packaging functional properties; and (iii) a solvent that should be non‐toxic, safe, and not affect the quality of the final product (Ribeiro et al., 2021). Edible film formulations based on proteins have to undergo denaturation to unfold their molecular chains. This process usually involves the addition of compounds such as acids or bases, or it can comprise physical treatment such as heat. Depending on the thermal load applied, the natural shape of globular whey proteins alters by exposing previously hidden sulfhydryl groups (SH) and hydrophobic amino acid side chains to the surrounding solvent. These SH groups can then rapidly form new disulfide bonds within and between protein molecules. This process, along with various protein–protein interactions during heating, leads to the formation of protein aggregates, which are necessary for creating a firm and flexible edible protein film network (Krochta & Monahan, 1996; Lefèvre et al., 2005). Heat treatment is also used not only for protein denaturation but also for pasteurization or sterilization of edible film‐forming solutions, as well as a mechanism to increase the solubility (Sol) of materials in the solvent (Ansorena et al., 2011; Chhikara & Kumar, 2022; Hassan et al., 2018).

Ohmic heating (OH) technology is currently receiving attention from the food industry because liquids are heated rapidly and uniformly, and this technology provides technical benefits in itself: greater energy efficiency (by reducing the need for an external heat source) and precise temperature control (making it effective for pasteurization or other thermal processing applications), ensuring microbial safety while maintaining product quality (Sakr & Liu, 2014; Sastry & Barach, 2000). The principle behind OH technology lies in Joule's law, which involves internal energy dissipation by passing an alternating electrical current through a product, generating internal heat due to the electrical resistance of the product itself (Vicente & Castro, 2007). Heat, which was generated from direct conversion of electrical energy, allows faster and more uniform temperature rise compared to traditional heating methods, which rely on external heat transfer. The amount of heat generated in OH depends on the intensity of the electrical current and the electrical conductivity of the food. As the temperature rises, the electrical conductivity of the food product typically increases, further enhancing the heating efficiency. However, this also requires careful control to avoid temperature runaway effects (Souza, Cerqueira, Teixeira, et al., 2010). Recent studies have highlighted the effects of OH treatment on various film properties. Wang et al. (2022) reported that OH treatment on soy protein isolate films enhanced fluorescence intensity, average particle size, and the polydispersity index as the strength of OH increased. Additionally, applying an electric field strength of 9 V/cm nearly doubled the tensile strength (TS) of the films. Similarly, Tinoco et al. (2020) demonstrated that OH treatment reduced the swelling of keratin‐based films by 55%, indicating its potential to modify and enhance film properties effectively. In contrast, OH could promote molecular rearrangement and enhance cross‐linking with the biopolymer structure. Additionally, the presence of an electrical field could induce secondary effects, such as alteration of polymer interactions, contributing to tailored film properties.

The goal of this study was to produce two different edible films from second‐hand dairy industry waste—liquid acid whey permeate (AWP) and liquid acid whey protein concentrate (AWPC)—using two heating treatments: conventional heating (CH) and OH. Additionally, the study aimed to compare the effects of these pasteurization methods on the rheological and microbiological properties of the film‐forming solutions and physicochemical, mechanical, and surface properties of the produced edible films. This research was conducted to evaluate the impact of OH compared to CH on the attributes mentioned above for the first time on whey‐based edible films.

## 2 MATERIALS AND METHODS

### 2.1 Materials

Liquid AWPC and liquid AWP were acquired from dairy plant AB Kauno pienas (Kaunas, Lithuania). The AWPC (dry matter: 18.29%, proteins: 2.19%, fats: 0.19%, pH: 4.55) and AWP (dry matter: 1.67%, proteins: 0.25%, fats: 0.17%, pH: 4.46) were frozen at −18°C until use. Before preparation of film‐forming solutions, AWPC and AWP were thawed at 4°C for 24 h.

Glycerol (99% purity) as a plasticizer, surfactant Tween 80 (Quality 200), and sunflower oil were supplied by Sigma‐Aldrich (3050 Spruce Street, Saint Louis, MO, USA). Sugar beet pectin (DE: 54%) (Betapec RU 301, Herbstreith & Fox GmbH & Co, Neuenbürg, Germany) was used as a thickener.

Rennet cheese was supplied from cheese factory E‐piim (Poltsama, Estonia).

### 2.2 Film‐forming solutions and film preparation

Films were produced by solvent evaporation technique according to Ramos et al. (2012) with some modifications. First, glycerol, sugar beet pectin, sunflower oil, and Tween 80 were mixed with AWPC in a glass beaker, and solution was warmed up for 5 min at 25°C in a water bath (Grant Instruments, OLS200, Cambridge, United Kingdom) to increase the Sol of solid materials and the solution was homogenized (stainless steel rotor 10 mm) (Ultra‐Turrax T18, Janke & Kunke, Staufen, Germany) at 15,000 rpm for 5 min. AWP film‐forming solutions were produced using the same methodology without sunflower oil. For OH application, mixtures of film‐forming solutions were transferred to a glass cylinder containing stainless steel electrodes from each edge. For CH, film‐forming solutions were transferred to glass storage bottles. After application of CH and OH treatments on film‐forming solutions, as described in Section 2.3, 30 g of each mixture were poured into 150 mm Petri dishes and dried for 12 h at 37°C in an oven to produce films. After the drying process, Petri dishes containing the formed films were closed, wrapped in Parafilm, and kept in desiccator until further analysis. The compositions of AWPC‐ and AWP‐based film‐forming solutions are presented in Table 1.

**TABLE 1:** Composition of liquid acid whey permeate (AWP) and liquid acid whey protein concentrate (AWPC) film‐forming solutions.

Film compound	AWPs^CH^ or AWPs^OH^	AWPCs^CH^ or AWPCs^OH^
Film basis (% w/w)	92.8	87.8
Sunflower oil (% w/w)	–	5
Sugar beet pectin (% w/w)	2	2
Glycerol (% w/w)	5	5
Tween 80 (% w/w)	0.2	0.2

*Note*: AWPs^CH^, AWPs^OH^: conventional and ohmic heating–treated liquid acid whey permeate film‐forming solutions, respectively; AWPCs^CH^, AWPCs^OH^: conventional and ohmic heating–treated liquid acid whey protein concentrate film‐forming solutions, respectively.

### 2.3 Heating treatments applied to film‐forming solutions

CH treatment was performed using a water bath (Grant Instruments, OLS200, Cambridge, United Kingdom) for film‐forming solution pasteurization. A glass storage bottle containing the AWPC or AWP film solutions was placed on a water bath, and samples were treated for a total of 50 min—45 min for heating up the solutions until reaching 95°C and holding at 95°C for 5 min—under constant agitation using a magnetic stirrer. The temperature during the heating process was controlled using a digital thermometer placed in the center of the film‐forming solutions.

OH treatment was performed in a glass cylinder containing a stainless steel electrode at each edge for film‐forming solution pasteurization as described by Pereira et al. (2010). First, film‐forming solutions electrical conductivity was measured using a bench conductivity meter (HI2003‐02, Hannah Instruments, Portugal) to assess the possibility of applying OH treatment for both solutions. AWPC and AWP expressed values of 5.25 and 6.47 mS/cm, respectively, which allowed studying the effect of OH on this type of film solutions. The OH treatments were performed for a total of 7 min—that is, 2 min of heating time to reach 95°C and 5 min holding time at 95°C. The function generator (1 Hz to 25 MHz and 1–10 V; Agilent 33220A, Penang, Malaysia) was used to regulate voltage output to keep temperature uniform during treatment time and then amplified on an amplifier system (Peavey CS3000, Meridian, MS, USA). The electric fields used ranged from 4.5 to 6.5 V/cm (during holding time at constant temperature) and were of 15 V/cm to achieve target temperature of 95°C. Type K thermocouples (Omega Engineering, Inc., Stamford, CT, USA) connected to a data logger (USB‐9161, National Instruments Corporation, Austin, TX, USA) were used to measure temperature. To ensure uniform heat distribution in the solutions during treatment, samples were gently stirred using a magnetic stirrer.

After both heating treatments, film‐forming solutions were left to cool down at room (20°C) temperature. Before and after both heating treatments, samples were transferred into sterile 2 mL Eppendorf's under aseptic conditions for further evaluation of pasteurization effectiveness.

### 2.4 Characterization of film‐forming solutions

#### 2.4.1 Pasteurization effectiveness of heating treatments

The pasteurization effectiveness of CH and OH treatments was assessed by total viable bacterial counts using the pour plate technique and expressed as log_10_ CFU/mL. First, film‐forming solution samples were taken before and after both heating treatments, followed by 10‐fold serial dilutions in buffered peptone water (Liofilchem, Italy). Total viable aerobic bacterial counts were enumerated on plate count agar (Oxoid, England) in triplicate and incubated at 30°C for 48 h according to ISO 4833‐1:2013 (International Organization for Standardization, 2013). Lactic acid bacteria (LAB) counts were enumerated on De Man–Rogosa–Sharpe agar (Oxoid, England) and incubated at 30°C for 72 h according to ISO 15214:1998 (International Organization for Standardization, 1998). Yeasts and molds were enumerated on potato dextrose agar (Oxoid, England) at 25°C for 120 h according to ISO 6611:2004 (International Organization for Standardization, 2004).

#### 2.4.2 Rheological properties

Rheological properties of film‐forming solutions after different heating treatments were obtained using a rheometer (Instruments HR1) equipped with a Peltier plate (TA Instruments, New Castle, NSW, USA) with a stainless steel cone–plate geometry (6.0 cm, 2° angle, truncation 67 µm). Measurements were performed at 25°C. Curves were obtained by the up‐down‐up step program using different shear stresses to provide shear rates ranging from 0 to 300 s^−1^. Rheological properties of film‐forming solutions were calculated using Newtonian (Equation 1) and power law (Equation 2) models:

(1) σ=η×γ

(2) $$\sigma =k\times \gamma^{n}$$

where *σ* is the shear stress (Pa), *η* is the viscosity (Pa s), *k* is the consistency index (Pa s*ⁿ*), *γ* is the shear rate (s^−1^), and *n* is the flow index.

#### 2.4.3 Contact angle measurements

The contact angle (CA) of film‐forming solutions with the intention to use them as a coating on a food product was determined using a CA meter (OCA 20, Dataphysics, Germany). A 500‐µL capacity syringe with a needle of 0.75 mm was used to dispense the film‐forming solution onto the Rennet cheese surface by the sessile drop method. Each measurement was obtained after 10 s at 20°C.

### 2.5 Characterization of the produced films

#### 2.5.1 Moisture content

A gravimetrical method was used to determine the moisture content (MC) in the films according to Coelho et al. (2017). Film samples with a 2 cm diameter were dried in an oven at 105°C for 24 h. The final calculations were done according to the following equation:

(3) $$\mathrm{Moisture}\;\mathrm{content}\;\left(\%\right)=\frac{X_{i}-X_{y}}{X_{i}}\times 100$$

where *X_i_* is the initial mass of the film sample, and *X_y_* is the mass of the dried film sample. The calculation is expressed as the percentage of water removed from the initial sample mass.

#### 2.5.2 Solubility

A circular film sample (2 cm diameter) was cut out, placed in the oven to dry at 105°C for 24 h, and weighed (Cuq et al., 1996). Then, 50 mL of water was poured on the samples. The samples were sealed with Parafilm and stirred in a shaker at 60 rpm for 24 h at 20°C. The non‐soluble parts of the films were taken out, dried at 105°C for 24 h, and then weighed to determine the weight of dry matter. The film Sol was determined using the following equation:

(4) $$\mathrm{Sol}\;\left(\%\right)=\frac{X_{i}-X_{y}}{X_{i}}\times 100$$

where *X_i_* and *X_y_* are the initial and final mass of the film sample, respectively.

#### 2.5.3 Water vapor permeability

The water vapor permeability (WVP) of the films was determined following the ASTM E96‐92 method with some modifications (Bourtoom & Chinnan, 2008). The film sample was sealed on top of a permeation cell filled with distilled water (100% relative humidity [RH]; 2.337 Pa vapor pressure at 20°C), and the cell was placed in a desiccator with silica gel at 20°C and 0% RH (0 Pa water vapor pressure). Over 10 h, the cups were weighed every 2 h. The weight loss of the permeation cell was used to calculate the amount of water that was absorbed by the desiccant and transported through the film. A tiny fan inside the desiccator maintained a steady air circulation outside the test cup, ensuring steady‐state and uniform water pressure conditions. The slope of the weight loss against time curve was determined using squares regression analysis.

#### 2.5.4 Film thickness

A hand‐held digital micrometer (No. 293–561, Mitutoyo, Japan) was used to determine film sample thickness. Measurements on testing samples were chosen from three random locations of each film. The mean values of thickness measurements were used for WVP and mechanical property calculation.

#### 2.5.5 Optical properties

The film color was determined using a digital Minolta colorimeter (Konica Minolta, Chroma meter CR‐400, Osaka, Japan). The CIELab system was used to determine color L*, a*, and b* coordinates—L* = 0 (black) to 100 (white); a* = −60 (green) to +60 (red); and b* = −60 (blue) to +60 (yellow). A white plate (calibration plate CX0384, L* = 92.82, a* = −1.24, and b* = 0.5) was used as a standard.

The opacity (*Y*) is expressed as a relationship between film opacity on a black standard (*Y_b_*) and the opacity of the white standard (*Y_w_*) and calculated according to the following equation:

(5) $$Y\;\left(\%\right)=\frac{Y_{b}}{Y_{w}}\times 100$$

#### 2.5.6 Mechanical properties

Elongation at break (EB) and TS of film samples (120 × 20 mm^2^) were measured using a TA.HD plus Texture Analyzer (Serial RS232, Stable Micro Systems, Surrey, UK), with a load cell of 5 kg, following the guidelines of ASTM D 882‐02.

#### 2.5.7 Scanning electron microscopy

The film sample morphology was observed using a Mira3 scanning electron microscope developed by Tescan Orsay Holding, a.s. (Brno‐Kohoutovice, Czech Republic). The samples were manually cut into 0.4 × 0.4 cm^2^ pieces and mounted on a 51 mm diameter silicon wafer (Micro to Nano, Haarlem, The Netherlands) without double‐sided adhesive carbon discs. Silicon was chosen as the substrate due to the prevalence of CHON elements in organic samples, eliminating elements that can bias the results and increase the signal‐to‐noise ratio. The scanning electron microscopy (SEM) was operated in high vacuum mode using backscattered electron and secondary electron detectors. Magnification was increased to 1.0 kx for accurate dimensional measurements and element composition analysis at 5 kV acceleration voltage.

#### 2.5.8 Energy‐dispersive x‐ray spectroscopy

Elemental analysis was performed by using an energy‐dispersive x‐ray spectrometer (EDS) equipped with an INCA x‐act LN2‐free analytical silicon drift detector manufactured by Oxford Instruments Inc. (Bognor Regis, UK), with a working distance of 15 mm to obtain energy spectrum hoarding. Imaging analysis was performed by collecting data from selected film regions (0.7 × 0.7 mm^2^) to analyze elemental distribution.

### 2.6 Statistical analysis

Statistical analysis was performed with the SPSS 23 statistical package (SPSS Inc., Armonk, NY, USA). Analysis of variance and Tukey's test of multiple comparisons with a significant level (α) of 0.05 were applied to evaluate the differences between film sample properties. All visuals were generated using Microsoft Excel 2021 package. Measurement values were expressed as the mean + standard deviation of at least three replicates.

## 3 RESULTS AND DISCUSSION

### 3.1 Pasteurization effectiveness of heating treatments

The impact of different heating treatments on the microbial counts in AWP and AWPC film‐forming solutions is presented in Table 2. Among all tested microbial groups, untreated film‐forming solutions exhibited the highest microbial counts. The AWPCS solution showed significantly higher amounts of LAB (3.96 log_10_ CFU/mL, *p* < 0.05) and total viable aerobic bacteria (3.84 log_10_ CFU/mL, *p* > 0.05), whereas the AWP film‐forming solution had lower counts of yeasts and molds (2.10 log_10_ CFU/mL, *p* > 0.05). These results indicate that the AWPC film‐forming solution had a greater initial bacterial load. After both CH and OH treatments, the microbial counts were below detection limits, showing that both pasteurization methods are promising in ensuring microbial safety of whey‐based film‐forming solutions. In addition to this, the presence of an electric field in OH results in minimal non‐thermal cellular damage. When a low‐frequency alternating current (<60 Hz) is applied, charges accumulate, leading to pore formation in cell walls, which could contribute to a higher increase of microbial cell death (Sain et al., 2024). In the study, conducted by Kim and Kang (2015), authors found that OH was capable of reducing pathogenic bacteria counts under detection limits more effectively and required less time than CH, emphasizing potential benefits of OH technology.

**TABLE 2:** Effect of different heating methods on microbial counts in whey‐based film‐forming solutions.

Film‐forming solution	Lactic acid bacteria count (log_10_ CFU/mL)	Total viable aerobic bacteria count (log_10_ CFU/mL)	Yeast and mold count (log_10_ CFU/mL)
AWPs	2.98 ± 0.03^a^	4.02 ± 0.11^a^	2.10 ± 0.17^a^
AWPCs	3.96 ± 0.17^b^	3.84 ± 0.20^a^	2.20 ± 0.17^a^
AWPs^CH^	<DL	<DL	<DL
AWPs^OH^	<DL	<DL	<DL
AWPCs^CH^	<DL	<DL	<DL
AWPCs^OH^	<DL	<DL	<DL

*Note*: AWPs, AWPCs: untreated liquid acid whey permeate and liquid acid whey protein concentrate film‐forming solutions, respectively; AWPs^CH^, AWPs^OH^: conventional and ohmic heating–treated liquid acid whey permeate film‐forming solutions, respectively; AWPCs^CH^, AWPCs^OH^: conventional and ohmic heating–treated liquid acid whey protein concentrate film‐forming solutions, respectively; <DL—below detection limit. Different lowercase letters in the same column indicate a significant difference (*p* < 0.05).

Abbreviations: AWPCs, acid whey protein concentrates; AWPs, acid whey permeates.

In contrast, both CH and OH treatments effectively achieved microbial inactivation. The absence of detectable microbial counts in OH‐treated film solutions indicates that this method is as effective as CH. However, OH technology provides a reduced risk of localized overheating due to its uniform heating within the solution, in that way enhancing microbial inactivation while minimizing the impact on heat‐sensitive compounds such as proteins and bioactive components (Varghese et al., 2014).

### 3.2 Rheological behavior of film‐forming solutions

**FIGURE 1:**
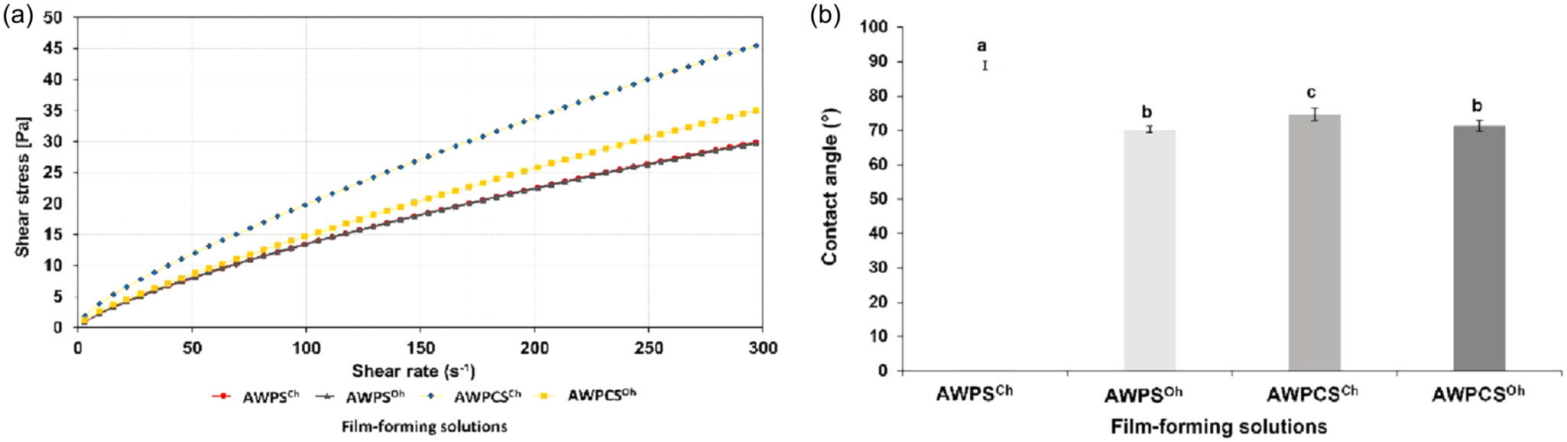
Rheology properties (a) and contact angle (b) of film‐forming solutions affected by different heating treatments. AWPs^CH^, AWPs^OH^: conventional and ohmic heating–treated liquid acid whey permeate film‐forming solutions, respectively; AWPCs^CH^, AWPCs^OH^: conventional and ohmic heating–treated liquid acid whey protein concentrate film‐forming solutions, respectively. Different lowercase letters mean a significant difference among samples (*p* < 0.05).

Rheological results (Figure 1a) demonstrated that different film‐forming solutions expressed non‐Newtonian, shear time‐independent behavior as *n* < 1 (Table 3), which implies a decrease in the apparent viscosity at increased shear rates. In addition, different heating methods did not affect the rheological properties of AWP film‐forming solution. Interestingly, the AWPC film‐forming solution treated by OH showed a slightly different behavior than the one treated by CH. The viscosity of the AWPC film‐forming solution treated with OH was lower than AWPC film‐forming solution treated with CH. Moreover, the shear stress of AWPC film‐forming solution treated with OH was similar to AWP film‐forming solutions up to a shear rate of 50 s^−1^. Despite the heating treatment applied, AWPC film‐forming solutions expressed higher viscosity than AWP. This result indicated that aggregates formed during OH can be altered in the structure during heating, impacting the consistency of the final film. Moreover, longer heating time during CH to reach desired temperature could affect protein aggregation, thus leading to higher viscosity. Moreover, OH, as an electrical heating method, can potentially lead to unexpected changes in the rheological properties of whey products and may include chemical reactions influenced by electrical effects, which can alter the consistency of whey solutions during ohmic processing (Costa et al., 2018; Icier, 2009). Chemical reactions may depend on the equipment used, such as electrochemical reactions occurring at the electrode–solution interfaces due to the use of stainless steel electrodes (Assiry et al., 2003; Samaranayake & Sastry, 2005). Because fouling and electrochemical reactions could not be ruled out, the possibility of these mechanisms leading to a lower viscosity observed in AWPC solution under OH treatment compared to CH at higher shear rates should not be rejected.

**TABLE 3:** Rheological properties of whey‐based film‐forming solutions affected by different heating treatments.

Film‐forming solution	*k*	*n*	*R* ^2^	*γ* (s^−1^)		
				3	153	297
				*η* (Pa s)		
AWPs^CH^	0.448	0.739	0.999	0.32 ± 0.02^a^	0.11 ± 0.01^a^	0.10 ± 0.01^a^
AWPs^OH^	0.464	0.732	0.999	0.32 ± 0.02^a^	0.12 ± 0.01^a^	0.10 ± 0.01^a^
AWPCs^CH^	0.699	0.732	0.999	0.60 ± 0.01^b^	0.17 ± 0.01^b^	0.15 ± 0.01^b^
AWPCs^OH^	0.418	0.777	0.999	0.37 ± 0.01^c^	0.13 ± 0.01^a^	0.11 ± 0.01^a^

*Note*: AWPs^CH^, AWPs^OH^: conventional and ohmic heating–treated liquid acid whey permeate film‐forming solutions, respectively; AWPCs^CH^, AWPCs^OH^: conventional and ohmic heating–treated liquid acid whey protein concentrate film‐forming solutions, respectively; *η*—viscosity (Pa s) at shear rates of 3, 153, and 297 s^−1^, *k*—consistency index (Pa s*ⁿ*), *γ*—shear rate (s^−1^), and *n*—flow index. Different lowercase letters indicate significant differences in the same column (*p* < 0.05).

Abbreviations: AWPCs, acid whey protein concentrates; AWPs, acid whey permeates.

### 3.3 Contact angle of film‐forming solutions on cheese surface

Results of CA of film‐forming solutions on cheese surface are presented in Figure 1b. Both AWP and AWPC film‐forming solutions after OH treatment expressed lower CA values than the solutions treated with CH, with differences between samples up to 18.53° and 3.25°, respectively (*p* < 0.05). The results indicated that CA values were higher for the AWP film‐forming solution, which was attributed to the CH effect, compared to the AWPC treated by OH (*p* < 0.05). The lower CA values, indicating better spreadability of film‐forming solutions over the cheese surface, are influenced by various factors. Among these, the properties of the droplet, such as the interactions between the film‐forming solution and the solid matrix, play a significant role (Zhong et al., 2014). The cheese surface, described as low‐energy with a higher dispersive component, likely participates in interactions characterized by dispersion forces. Given cheese's richness in apolar components, it exhibits apolar features that contribute to these interactions (Cerqueira et al., 2009). It seems that OH affected film‐forming solutions by inducing phase transitions by melting and increasing crystallization.

### 3.4 MC, Sol, thickness, and WVP of produced films

The MC of the edible films is an important parameter that can influence their properties. For example, higher MC of the films could contribute to microbial spoilage of the food product and even affect the durability of the films produced (Apriliyani et al., 2020). In the present study, no MC statistical differences were found between the same type of films treated with different heating methods (*p* > 0.05), as can be seen in Table 4. However, significant MC differences were observed between AWP and AWPC films. The AWP films exhibited higher MC than AWPC films (around 2%) despite the heating treatment applied (*p* < 0.05). Because heating treatments did not affect the MC of the films, the MC differences between films could be related to the type of whey used and sunflower oil addition into AWPC films. There is evidence that the oil addition to films reduced MC due to oil hydrophobic nature (Galus & Kadzińska, 2016).

**TABLE 4:** Moisture content (MC), solubility (Sol), water vapor permeability (WVP), and thickness of whey‐based films.

Film	MC (%)	Sol (%)	WVP (×10^7^, g/h/m/Pa)	Thickness (mm)
AWP^CH^	26.81 ± 0.88^a^	13.18 ± 0.18^a^	36.21 ± 0.42^a^	0.27 ± 0.00^a^
AWP^OH^	26.56 ± 0.26^a^	6.15 ± 0.65^b^	36.73 ± 0.69^a^	0.24 ± 0.01^b^
AWPCH^CH^	24.75 ± 0.39^b^	20.08 ± 0.92^c^	45.76 ± 3.85^b^	0.77 ± 0.01^c^
AWPC^OH^	24.80 ± 0.72^b^	24.97 ± 0.83^d^	45.44 ± 1.29^b^	0.64 ± 0.01^d^

*Note*: AWP^CH^, AWP^OH^: conventional and ohmic heating–treated liquid acid whey permeate films, respectively; AWPC^CH^, AWPC^OH^: conventional and ohmic heating–treated liquid acid whey protein concentrate films, respectively. Different lowercase letters mean a significant difference in the same column (*p* < 0.05).

Abbreviations: AWPC, acid whey protein concentrate; AWP, acid whey permeate.

Sol is a critical parameter of edible films because it generally indicates the film's resistance to water and thus determines barrier properties and preservation capacity of produced films. Water‐resistant films are more suitable for the protection of products with high water activity (Sébastien et al., 2006; Souza et al., 2009). In the present study, OH‐treated AWP films showed a reduction of Sol compared to CH‐treated AWP films. On the other hand, Sol of OH‐treated AWPC films increased compared to CH‐treated AWPC films (*p* < 0.05). Moreover, films produced from AWPC demonstrated higher Sol than AWP (*p* < 0.05). Souza et al. (2009) found that OH interfered with the degree of crystallinity, which facilitated hydrogen bonding in the films, reducing film Sol in water. Moreover, Sol of films made from different whey‐based materials could be dependent on the aggregates that were formed during the heating process. OH could influence aggregates’ shape and size and interfere with protein aggregation pathways, thus resulting in a change of behavior of aggregates due to developed properties in the process affecting the Sol (Pereira et al., 2016).

WVP shows the ability of the film to prevent moisture loss in packaged foods. Water vapor can be transferred from the internal or external surroundings of the food, which could result in a decrease of product quality and shelf life (Ballesteros‐Mártinez et al., 2020; Caetano et al., 2017). Results showed that CH and OH treatments did not significantly affect the WVP of the whey‐based films (*p* > 0.05). However, films produced from AWPC demonstrated lower WVP than AWP (*p* < 0.05). de Vargas et al. (2022) found that OH reduced the WVP of gelatin‐based films compared to those treated with CH. This reduction was likely due to both the thermal and non‐thermal effects of OH, as the applied electric field can create a more ordered structure within the film, reducing amorphous zones through which water vapor could permeate. However, Wang et al. (2022) observed that OH exposure could also lead to protein aggregation in films. They noted that if higher electric field intensity is applied, this aggregation may result in film defects, indicating that the effects from the electric field need careful control. As we did not find any bulky aggregates in CH‐ and OH‐treated films by SEM analysis, this might explain the absence of significant differences between the same type of films treated differently.

Film thickness is an essential physical property that affects the biological and physical properties and shelf life of the coated material or product (Kocira et al., 2021). In the present study, OH treatment reduced AWP and AWPC film thickness compared to CH treatment (*p* < 0.05). In addition, AWP films showed lower thickness values than AWPC films, despite the heating treatment used (*p* < 0.05). These findings aligned with previous studies that have demonstrated that films undergo a reduction in thickness after OH application, likely attributed to protein aggregation at some degree during the heating process (Pereira et al., 2010; Wang et al., 2022).

### 3.5 Optical properties of films

L* values of films did not change significantly despite the heating treatment used (*p* < 0.05), as can be observed in Table 5. Both films’ a* values were affected by OH, where AWP films showed a decrease of a* values, whereas AWPC films demonstrated an increase of a* values. On the other hand, OH treatment increased the b* values of AWP films and reduced the b* values of AWPC films. Finally, it was noted that AWP films were less opaque than AWPC films (Table 5). The differences observed could depend on the dry basis matter of the films. Previous research indicated that color changes may occur in milk products during heating and can depend on the product's pH. Li‐Chan (1983) stated that color changes may depend on the Maillard reaction, as it was found that samples that showed darker colors presented higher pH values, as the Maillard reaction is favored in more alkaline environment. If such a reaction occurred in the current study during heating treatments, it is possible that irregular tendencies arose, particularly because samples subjected to CH required more time to reach the desired temperature. Additionally, more studies are needed regarding different heating methods and their effects on color parameters of films produced from whey to evaluate the mechanism responsible for changes in product's color.

**TABLE 5:** Optical properties of whey‐based films treated with different heating treatments.

Film	L*	a*	b*	Opacity (%)
AWP^CH^	91.17 ± 0.33^b^	−1.43 ± 0.09^a^	12.81 ± 0.72^a^	12.36 ± 0.18^a^
AWP^OH^	89.14 ± 0.76^b^	−1.91 ± 0.13^b^	17.00 ± 1.56^b^	15.10 ± 0.56^b^
AWPC^CH^	81.50 ± 1.65^a^	−3.56 ± 0.02^c^	26.40 ± 2.16^c^	26.09 ± 1.03^c^
AWPC^OH^	83.11 ± 0.56^a^	−2.75 ± 0.10^d^	18.38 ± 0.41^b^	23.02 ± 0.90^d^

*Note*: AWP^CH^, AWP^OH^: conventional and ohmic heating–treated liquid acid whey permeate films, respectively; AWPC^CH^, AWPC^OH^: conventional and ohmic heating–treated liquid acid whey protein concentrate films, respectively. Different lowercase letters mean significant difference in the same column (*p* < 0.05).

Abbreviations: AWPC, acid whey protein concentrate; AWP, acid whey permeate.

### 3.6 Mechanical properties of the films

**FIGURE 2:**
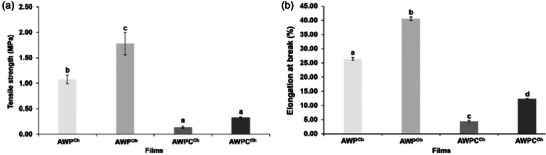
Mechanical properties—tensile strength (a) and elongation at break (b)—of produced whey‐based films. AWP^CH^, AWP^OH^: conventional and ohmic heating–treated liquid acid whey permeate films, respectively; AWPC^CH^, AWPC^OH^: conventional and ohmic heating–treated liquid acid whey protein concentrate films, respectively. Different lowercase letters mean a significant difference between samples (*p* < 0.05).

Films treated with OH presented TS and EB values significantly higher than films treated with CH, indicating a positive effect of the electrical fields on the film mechanical properties (Figure 2). Among all produced films, AWP films treated with OH expressed the highest TS (1.78 MPa) and EB (40.61%) values. Rodrigues et al. (2015) stated that the improvement of mechanical properties of films could be due to the involvement of the proteins in the matrix because the application of an electrical field can cause the disturbances in protein structures, which is probably caused by alternating movement of electrical charges. This movement could induce interaction between molecules of proteins, including hydrophobic and electrostatic interactions. Souza et al. (2010) found evidence of the positive effect of applying electrical field treatment, where treated films expressed 9% higher values of TS and 18% higher values of EB than untreated samples. The authors attributed these results to a consequence of an increase of the film crystallinity. Similar findings on enhanced mechanical properties due to increased crystallinity in films can be found in a study conducted by Bigi et al. (2004), where the authors investigated gelatin films’ triple‐helix content and mechanical properties. In addition to this, it is essential to consider the time required to process each film‐forming solution. Prolonged exposure of film‐forming solution to CH could have led to thermal degradation of proteins and other structural components, consequently resulting in a negative impact of the films’ mechanical properties. Longer film‐forming solution exposure to heat during CH processing potentially could lead to weaker or more brittle films. While analyzing the surface hydrophobicity and mechanical properties of soy protein isolate films, Wang et al. (2022) found that the thermal effect of CH treatment was stronger than that of OH. The excessive heating in CH led to protein aggregation, which may have contributed to a reduction in TS in the treated films.

### 3.7 Microstructure of the films

**FIGURE 3:**
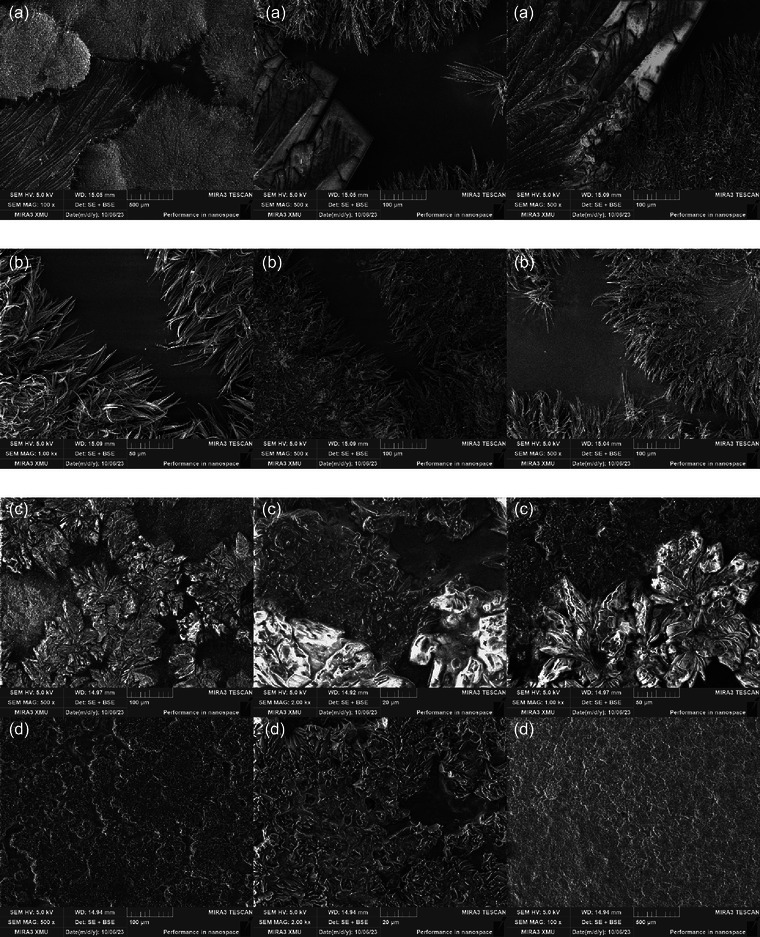
Scanning electron microscopy images of the surface of films produced using different heating treatments: (a) conventional and (b) ohmic heating–treated liquid acid whey permeate films, respectively; (c) conventional and (d) ohmic heating–treated liquid acid whey protein concentrate films, respectively.

Overall, CH and OH treatments had an effect on film microstructure (Figure 3). AWP and AWPC films treated with CH showed rigid and various forms of crystals with large spaces between them in the film surface. On the other hand, AWP and AWPC films treated with OH showed a uniform crystal cluster formation with smaller spaces between crystals throughout entire film surface. Analysis of SEM micrographs revealed that OH affected the film structure uniformly, allowing the formation of homogenous crystal network across the entire film surface during the drying process. This suggests that crystal formation is an important factor as it can affect various film properties, such as WVP, TS, and EB (Iahnke et al., 2019; Pereira et al., 2010; Souza, Cerqueira, Martins, et al., 2010). Moreover, SEM images revealed that CH or OH treatments did not affect film formation negatively, as it was possible to observe no pores or holes in the films during the drying process. After studying the effects of different heating methods, Lei et al. (2007) observed that OH offers an advantage by dispersing the heat uniformly throughout the entire liquid compared to CH. Balau et al. (2004) demonstrated that electric fields play an important role in the formation of a crystal network. In their study, films treated with an electric field of 20 kV/cm formed a crystalline structure, whereas no‐treatment films contained significantly less crystalline material. In addition, the uniformity of crystal formation with smaller and tightly packed crystals can increase the overall mechanical strength of the films by improving the internal cohesion of the matrix. The passage of electric current in OH not only induces uniform heat distribution but also promotes molecular interactions and reorganization within the film matrix. Moreover, electric field can induce conformation changes and encourage crosslinking, leading to a more ordered and tightly packed structure (Jiang et al., 2019; Shah et al., 2023).

### 3.8 Elemental composition analysis of the films

**FIGURE 4:**
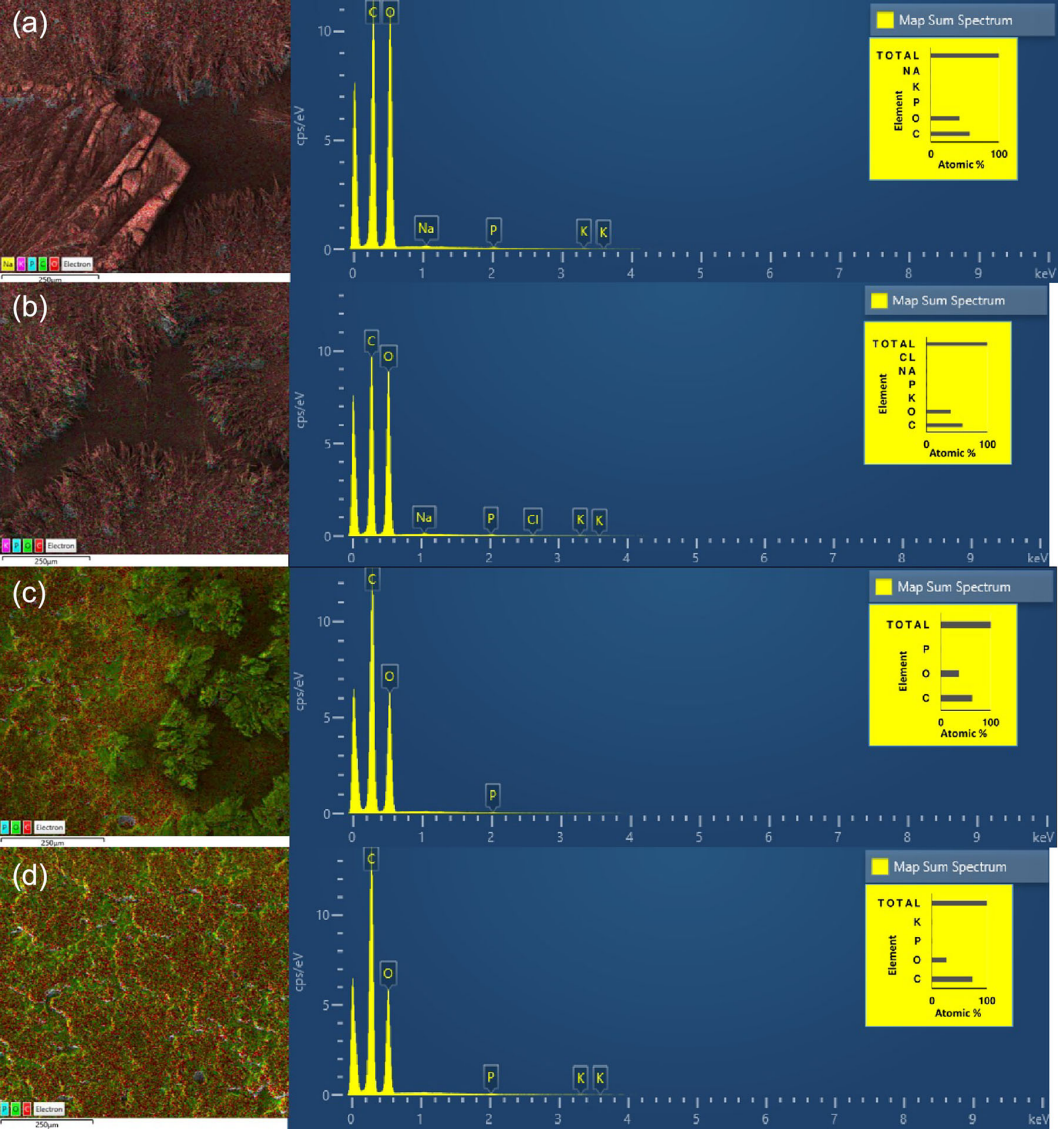
Energy‐dispersive x‐ray spectroscopy spectra of (a) conventional and (b) ohmic heating–treated acid whey permeate films, respectively; (c) conventional and (d) ohmic heating–treated liquid acid whey protein concentrate films, respectively.

EDS spectra of the whey‐based films are presented in Figure 4. No significant differences were detected between the same type of films. Cl element was detected after OH treatment on AWP films (slight peak) along with a slight increase of C and a slight decrease of O elements. The same tendency was observed in AWPC films, where a slight K peak was detected after OH treatment. Furthermore, elements were evenly distributed across the OH‐treated film surface compared to the CH‐treated film surface, where elements were clustered in one region. This tendency could be related to crystal formation and chemical bonding, with element clusters concentrated in crystal regions. Literature states that the most abundant elements in organic matter are C and O, with low levels of other elements whose content mostly depends on the production and impurities of the materials used (A.g et al., 2017; Murrieta‐Pazos et al., 2013). To our knowledge, there are no studies on the effect of different heating methods on the element distribution in films produced from AWP and AWPC; thus, further studies are needed to understand this mechanism.

## 4 CONCLUSION

The comprehensive analyses of how film composition and processing methods affect the properties of edible films highlight the importance of considering their optimization for diverse food applications. CH and OH proved to be effective pasteurization methods for preventing microbiological growth in whey‐based film‐forming solutions. Mechanical properties like TS and EB were influenced by both film composition and heating treatment methods. Notably, electrical field treatments showed promising outcomes in enhancing the mechanical properties of produced films, inspiring potential advancements in film technology. On the other hand, Sol of films showed varied responses to different heating methods, highlighting the intricate interplay between film composition and heating processing. Furthermore, it was observed that OH influenced crystal formation during the drying process of the films by forming clusters on the film surface. Elemental composition analysis revealed subtle differences in elemental distribution post‐treatment, suggesting potential implications for film properties. Overall, these findings contribute to advancing our understanding of whey‐based edible film properties and provide insights for future research to explore the mechanisms underlying their behavior under different heating treatment methods.

## AUTHOR CONTRIBUTIONS

**Elvidas Aleksandrovas**: Conceptualization; data curation; formal analysis; investigation; visualization; writing—original draft. **Agnė Vasiliauskaitė**: Formal analysis; investigation. **Jorge M. Vieira**: Methodology; investigation; writing—review and editing. **Joana T. Martins**: Investigation; methodology; writing—review and editing. **Ricardo N. Pereira**: Investigation; methodology; writing—review and editing. **Antonio A. Vicente**: Methodology; supervision; writing—review and editing. **Vitalijs Radenkovs**: Methodology; software; writing—review and editing. **Ida Rud**: Writing—review and editing. **Mindaugas Malakauskas**: Funding acquisition; resources; validation. **Loreta Šernienė**: Conceptualization; project administration; supervision; writing—review and editing.

## CONFLICT OF INTEREST STATEMENT

The authors declare no conflicts of interest.
